# Terbinafine as a successful treatment in primary cutaneous aspergillosis^⋆^

**DOI:** 10.1016/j.abd.2023.07.011

**Published:** 2024-04-22

**Authors:** Juan-Manuel Morón-Ocaña, Isabel-María Coronel-Pérez, Elena-Margarita Rodríguez-Rey

**Affiliations:** Department of Dermatology, Virgen de Valme Hospital, Sevilla, Spain

Dear Editor,

Aspergillus is a ubiquitous saprophytic mold in nature and is commonly found in soil water and decaying vegetation. The most common human pathogens include *A. fumigatus* (85%), *A. flavus* (5%‒10%) and *A. niger* (2%‒3%).^1^

Aspergillosis usually occurs in immunocompromised hosts. Primary cutaneous aspergillosis (PCA) is a rare but life-threatening invasive fungal infection of the skin caused by *Aspergillus*. Due to its clinical heterogeneity, clinical suspicion should be high in immunosuppressed patients.^1^

The literature is replete with reports of PCA, however there is not a single reported case treated with terbinafine in monotherapy.

A 74-year-old man presented for evaluation of a mass in his right leg for a year. He had been under tacrolimus, prednisone, and mycophenolate mofetil treatment since 2012 because of a renal transplant. The patient denied any previous trauma, but he had presented a torpid venous ulcer in the area. Physical examination revealed violaceous and skin-colored subcutaneous nodules with superficial exulceration in the lower third of the right leg (Fig. 1). A skin biopsy was performed, and samples were sent to the pathology and microbiology labs.

**Figure 1:**
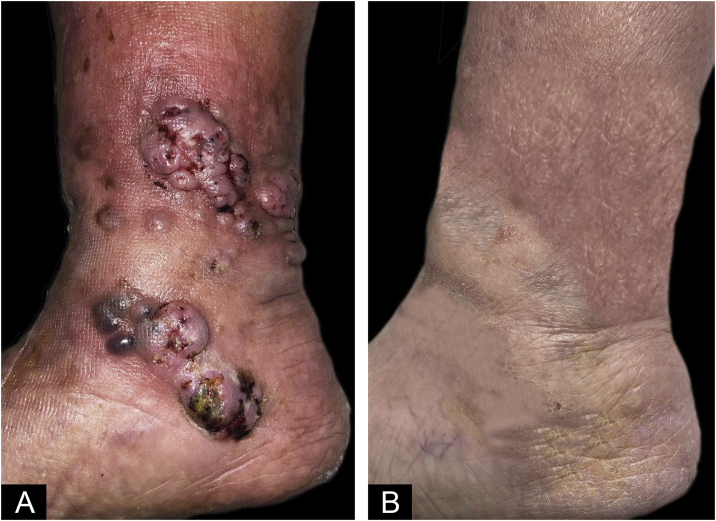
Physical exploration: (A) Initial physical exploration. Nodules with superficial exulceration in the lower third of the right leg of 0.5‒1 cm, coalescing with each other, forming 4‒5 cm plaques. (B) Physical exploration after 3-months of terbinafine. Residual hyperpigmentation on right left.

The skin biopsy showed septate hyphae with right angulation and vesiculation (Fig. 2A). These structures corresponded to the growth of colonies composed of *Aspergillus fumigatus* (Fig. 2B‒C). Blood cultures, galactomannan antigen test, and a chest-abdominal CT scan were performed. The results of all tests were negative. After rejecting systemic involvement, the patient was definitively diagnosed with PCA.

**Figure 2:**
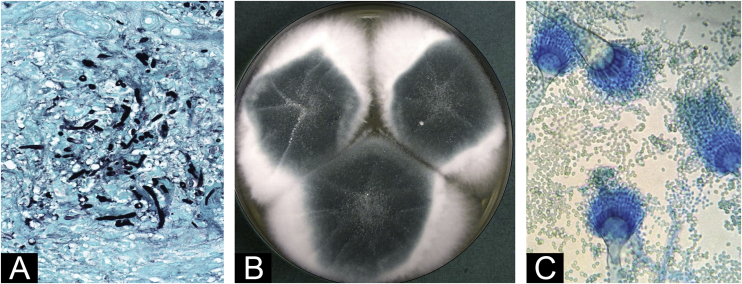
Supplementary tests: (A) Skin biopsy, (Groccot, 40×). Septate hyphae, with right angulation, vesiculation and pseudohyphae. (B) Microbiological culture of skin biopsy on dextrose agar. Colonies composed of a bluish-green central portion surrounded by a whitish foamy edge. (C) Lactophenol blue stained microscopy of microbiological culture. Septate hyaline hyphae were found with typical reproduction forms of *Aspergillus fumigatus*: aspergilate heads, with smooth and regular conidiophores, club-shaped vesicles covered in their upper third by uniseriad phialides that gave rise to smooth and globose conidia.

Oral isavuconazole was started but it was suspended because of an important elevation of tacrolimus plasma levels. After that, the patient started terbinafine 250 mg/24h. The lesions disappeared leaving only residual hyperpigmentation after 3 months of treatment (Fig. 1). The presence of two negative cultures separated from each other by 3 months confirmed the resolution of the infection.

Currently, there are four classes of antifungal agents with activity against *Aspergillus*: 1) The polyenes, such as amphotericin B deoxycholate and nystatin, 2) The triazoles, including itraconazole, voriconazole, isavuconazole; 3) The echinocandins, such as caspofungin and micafungin and 4) The allylamines such as terbinafine.^2^

Until the early 1990s, amphotericin B deoxycholate was the only agent that was available for the management of this infection. However, the significant toxicities associated with this agent made it less attractive with the introduction of newer agents such as the triazoles and the echinocandins, which are much better tolerated.^2^

Among these, isavuconazole proved to be superior in terms of response, toxicity and overall survival.^2^ However, triazoles have been found to have inhibitory effects on hepatic cytochrome P450.^3^ The inhibition of the cytochrome P450 can produce an important elevation of plasmatic levels of drugs that are metabolized by this route and can cause significant toxicities, as has happened with the tacrolimus that our patient was taking.

The other antifungals classically effective against *A. fumigatus* could also have had side effects on our patient. Amphotericin B deoxycholate is highly nephrotoxic and could have increased the nephrotoxicity in a kidney recipient transplant.^2^ On the other hand, using caspofungin together with tacrolimus may have decreased the plasma levels of tacrolimus,^4^ increasing the risk of kidney transplant loss.

Although it has been well-known for years that terbinafine is effective in vitro in aspergillosis, there are no published cases that have clinically demonstrated its efficacy in vivo. Importantly, because of its poor penetration in deep tissues, terbinafine is almost exclusively indicated for skin and nail infections.^2^ Schmitt et al. demonstrated that concentrations between 0.8‒1.6 μg/mL of terbinafine are sufficient to reach the Minimum Inhibitory and Fungicide Concentration (MIC and MFC) against *A. fumigatus* in a vitro study.^5^ As claimed by its fact sheet, a 250 mg single dose of terbinafine (standard dose marketed) is able to reach a serum concentration from 0.8 to 1.5 μg/mL two hours later after ingesting the pill.^6^ Terbinafine can also penetrate excellently from blood to skin according to its pharmacokinetics.^2^ As the concentration of terbinafine that is reached in plasma is very similar to the MIC of *Aspergillus*, terbinafine in monotherapy demonstrates good activity against *Aspergillus spp.* in the skin.^6^

Although further investigation is required, this unique case evidence that PCA could successfully be treated by terbinafine.

## Financial support

None declared.

## Authors’ contributions

Juan Manuel Morón Ocaña: Preparation and writing of the manuscript; critical literature review.

Isabel María Coronel Pérez: Approval of the final version of the manuscript; manuscript critical review.

Elena-Margarita Rodríguez Rey: Approval of the final version of the manuscript; manuscript critical review.

## Conflicts of interest

None declared.
